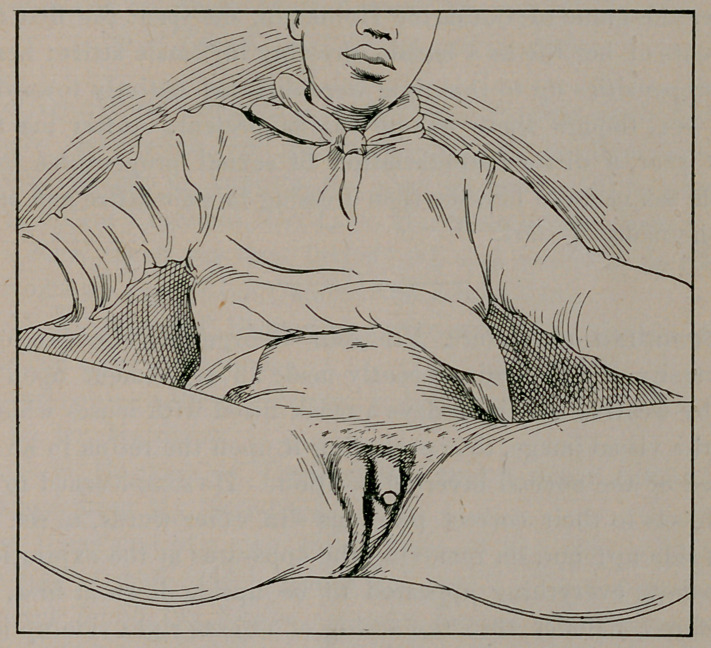# A Case of Presumable Hermaphrodism

**Published:** 1898-05

**Authors:** Edward Nicholas Liell

**Affiliations:** Jacksonville, Fla.; Late Lecturer on Gynecology, New York Polyclinic


### A CASE OF PRESUMABLE HERMAPHRODISM.

By EDWARD NICHOLAS LIELL, Jacksonville, Fla.
Late Lecturer on Gynecology, New York Polyclinic.

At the December meeting of the Duval County Medical Society I referred to the following interesting case of presumable hermaphrodism, the individual being a negro, at work in a convict camp in South Florida. The following is a description of the conditions presenting, accompanied by a photograph of the same. Age, twenty-two years; has always dressed in male attire, but has never been accustomed to hard work; has been a cook and waiter. Has a well developed penis in natural position, though rather small in size, being about the size of two joints of a man's finger. There is an opening, as of urethra, in the glans penis, but there is apparently no canal, the urine being voided through an opening at the base of the penis; the urine, when voided, takes a direction from the body just as if from the normal male urethra. About an inch below the base of the penis is a vaginal opening, which is quite small and short, barely admitting the tip of one's little finger. There is no scrotum present. The labia majora are, however, prominent, and are well shown on the accompanying illustration. Just to the right of the base of what may be called the penis, at the upper portion of the right labia majora, can be felt, freely movable, a small glandular body about the size of a pecan nut, and which, from its position in the immediate region of the inguinal opening, may be regarded as either an undeveloped testicle or an ovary in an abnormal situation. The individual claims to have felt sexual passion, with erection of the penis at such times, although intercourse has never been had. Slight "signs" of menstruation have also been claimed as having occurred at irregular intervals.

As shown by the history as above recorded, and made more evident by the illustration, the characteristics of both the male and the female sex are evidently united in this individual, those of the female predominating. I regret the fact that no examination was made as to the mammary glands. If possible, a more thorough examination will be made subsequent to release from State control.

In the *American Journal of Obstetrics*, February, 1876, is recorded by Mundē, with several illustrations accompanying and bearing upon the text, a rare case of presumptive true lateral hermaphrodism, the individual possessing also the characteristics of both the male and female sex united in his person. The individual was exhibited before the New York Obstetrical Society, October 5, 1875. Under the name of Catharine Hohmann, she spent the first forty-six years of her life as a female, dressing in female attire; her sexual propensities up to this time were directed entirely towards the male sex, though there was no vagina present. After her forty-sixth year, a distinct appreciation of sexual propensities for the female sex came to her, she then dressing in male attire and assuming the name of Carl.

---

ACCORDING to *Nature*, Professor George M. Stratton, of the University of California, recently made an experiment upon himself by wearing for eight days a mask fitted with lenses which invert the visual image, thus projecting it upon the retina in an erect instead of the normal inverted position. He soon learned to refer all objects to their correct positions—in other words, to see them right side up; but, on removing the apparatus at the expiration of eight days, everything appeared to be upside down at first. He therefore concludes that the seeing of objects right side up is due to a mental rectification of the visual image actually projected upon the retina.—*Phil. Med. Journal.*

---

When a patient comes to you with enlarged lymph nodes of the neck, be sure to examine the throat most carefully. If the patient is a child, remember that a very common cause of lymph node inflammation is the presence of hypertrophied tonsils or of adenoid vegetations. In an individual of middle age, examine any hypertrophy critically, bearing in mind the possibility of neoplasm.—*International Journal of Surgery.*

## Figures and Tables

**Figure f1:**